# Understanding collaboration in a multi-national research capacity-building partnership: a qualitative study

**DOI:** 10.1186/s12961-016-0132-1

**Published:** 2016-08-18

**Authors:** Dinansha Varshney, Salla Atkins, Arindam Das, Vishal Diwan

**Affiliations:** 1 Department of Public Health and Environment, RD Gardi Medical College, Ujjain, India; 2 Department of Public Health Sciences, Karolinska Institutet, Stockholm, Sweden; 3 Institute of Health Management Research University, Jaipur, India; 4 International Centre for Health Research, RD Gardi Medical College, Ujjain, India

**Keywords:** International collaboration, Research capacity building, Social determinants of health

## Abstract

**Background:**

Research capacity building and its impact on policy and international research partnership is increasingly seen as important. High income and low- and middle-income countries frequently engage in research collaborations. These can have a positive impact on research capacity building, provided such partnerships are long-term collaborations with a unified aim, but they can also have challenges. What are these challenges, which often result in a short term/ non viable collaboration? Does such collaboration results in capacity building? What are the requirements to make any collaboration sustainable? This study aimed to answer these and other research questions through examining an international collaboration in one multi-country research capacity building project ARCADE RSDH (Asian Regional Capacity Development for Research on Social Determinants of Health).

**Method:**

A qualitative study was conducted that focused on the reasons for the collaboration, collaboration patterns involved, processes of exchanging information, barriers faced and perceived growth in research capacity. In-depth interviews were conducted with the principal investigators (n = 12), research assistants (n = 2) and a scientific coordinator (n = 1) of the collaborating institutes. Data were analysed using thematic framework analysis.

**Results:**

The initial contact between institutes was through previous collaborations. The collaboration was affected by the organisational structure of the partner institutes, political influences and the collaboration design. Communication was usually conducted online, which was affected by differences in time and language and inefficient infrastructure. Limited funding resulted in restricted engagement by some partners.

**Conclusion:**

This study explored work in a large, North-South collaboration project focusing on building research capacity in partner institutes. The project helped strengthen research capacity, though differences in organization types, existing research capacity, culture, time, and language acted as obstacles to the success of the project. Managing these differences requires preplanned strategies to develop functional communication channels among the partners, maintaining transparency, and sharing the rewards and benefits at all stages of collaboration.

**Electronic supplementary material:**

The online version of this article (doi:10.1186/s12961-016-0132-1) contains supplementary material, which is available to authorized users.

## Background

Health research capacity can contribute to overall health system development of any country, particularly in low- and middle-income countries (LMICs), where there is less capacity in research [1, 2]. The reasons for low research output from LMICs can be shortages of local qualified researchers, limited funding, poor infrastructure, and lack of expertise in academic writing [3–7]. Building and sustaining research capacity within developing countries is a complex [7, 8], but essential and effective means of accelerating research contributions to health and development [9–12]. However, researchers have noted that 90% of global research investments addresses the needs of only 10% of the world’s population [13]. Most research output is also from high-income countries [14].

Facilitating collaboration between developed and developing counterparts [15] could result in higher research outputs from LMICs [16, 17]. To this end, organisations, such as the Council on Health Research for Development (COHRED) in 2003, were created at the global level to work directly with governments in LMICs to promote national and international collaborations [18]. Research collaboration in general has grown in importance for scientists, research organisations and policymakers [19–21]. However, in spite of many initiatives, generally originating from high-income countries, collaborations are often criticised for failing to strengthen, incorporate, and involve low-income partners in priority setting and publications, and research collaboration does not always result in increased outputs [15]. An international, cross-disciplinary, project faces many challenges, such as communication and coordination problems, misunderstandings, and mismatched expectations. Participants in such projects come from different fields of work and work for a unified goal, and are usually dependent on each other [16]. Therefore, the responsibility for making such collaboration successful falls onto the lead researchers, leading others at partner universities and institutes. This management of  multiple stakeholders with different resources and expectations is also a challenge [13]. It is suggested that successful research collaborations need exploration and identification of areas of interventions, effective dissemination strategies, uptake of results and, most importantly, the commitment of the partner countries [9, 10]. Barriers to successful collaboration include, amongst others, aims that are not shared, unequal distribution of power, lack of trust, ineffective membership structures and poor leadership [22].

Despite these challenges, international collaborations are often presented as a panacea for particularly complex issues and problems that exist within the fields of policy and politics in a wide range of international contexts [23]. Many benefits and requirements of such interventions are documented in the past, but the actual process of the collaboration has been studied infrequently. Given the challenges in trans-disciplinary and international collaborative research, and the possible barriers to success, it is important to learn from existing collaborations to give recommendations for countering challenges. Therefore, we aimed to understand (1) What are these challenges, which often result in a short-term/non-viable collaboration? (2) Does such collaboration result in capacity building? and (3) What are the requirements to make collaborations sustainable? in one multi-country research collaboration, ARCADE RSDH (Asian Regional Capacity Development Research on Social Determinants of Health).

## Methods

### ARCADE RSDH Project

ARCADE RSDH (www.arcade-project.org) was developed in response to inequities in health in the Asian context, coinciding with weak local health research capacity, especially in the social determinants of health (SDH) research [24]. The project originated as an adaptation of its sister project ARCADE HSSR. The project developed and progressed through funds from the European Union. The purpose of this collaboration was to add new research training capacity by training a new generation of researchers. The focus was on postgraduate, doctoral and postdoctoral training in LMICs in Asia, and on the promotion of research on the SDH [24]. Under this collaboration, many courses were developed and delivered across institutions, and institutional capacity in grants management and communications was built [25, 26]. Various innovative technologies were used by the project to produce world class online learning modules. Through innovative technology, courses were made available to researchers in LMICs that may not otherwise have had access to such material. The list of ARCADE partners is shown in Table 1.

**Table 1:** List of ARCADE RSDH Partners

S.no	Name of the participating organisation	Country’s name	Short names of the organisation
1	Karolinska Institutet	Sweden	KI (Coordinating institute)
2	Huazhong University of Science and Technology	China	TJMC (Hub institute)
3	CBCI Society of Medical Education	India	SJNAHS (Hub Institute)
4	Institute of Development Studies	United Kingdom	IDS (Big partner)
5	Beijing Normal University	China	BNU (Small partner)
6	Indian Institute of Health Management Research	India	IIHMR (Small partner)
7	University of Tampere	Finland	UTA
8	Zhejiang University	China	ZJU (Small partner)
9	Ujjain Charitable Trust Hospital and Research Centre	India	RDGMC (Small partner)
10	Sultan Qaboos University	Oman	SQU (Small partner)
11	Hanoi Medical University	Vietnam	HMU (Small partner)
12	Stellenbosch University	South Africa	SU (Small partner)

### The ARCADE Consortia

ARCADE RSDH operated through 12 universities operating within a network with expertise in research in social determinants of health or related areas. Karolinska Institutet (KI), based in Sweden, coordinated the project activities. At each partner institution, Principal Investigators (PI) were mostly senior staff, supported by junior staff (postdoctoral fellows, researchers or PhD students) in running the project.

Activities were developed collaboratively within the consortium. The consortium had two regional training centres (hubs; TJMC and SJNANHS, at the time of this study), each training doctoral and postdoctoral students, from their own countries, from the other Asian partners and from the European partners, as well as open to international student applications. The European partners and their networks supported the two regional training centres through staff visits and exchanges. The two regional training centres supported each other through exchange of course materials, experience, skills and staff, joint training programmes for their students, and joint applications for research and research training grants in cooperation with European partners.

### Study setting

We conducted a qualitative interview study online in R D Gardi Medical College, Ujjain, India with 12 partner universities, from India, China, Oman, Vietnam, UK, Sweden, Finland and South Africa, between March 2014 to June 2014. The study was conducted in the middle phase of this project. 

### Data collection

Email invitations were sent to all the PIs and project staff of the partner organizations for in-depth interviews, stating the purpose and objectives of the study. In total, 16 participants from 12 institutions (12 PIs, two research assistants, one project manager and one scientific coordinator) participated in the study. Half of the interviewees were key people involved in formulation and execution of the project, and the rest played a less central role. Individual interviews at a time and place convenient to the participants were conducted in English through Skype in March and April 2014. The interviews followed a set of topic guides, which were designed for the following categories of participants: coordinating organisation partners with large funding including the hub and partners with little funding. The topic guide addressed (1) the reasons for collaboration, (2) how the information was exchanged and the challenges faced while carrying out project’s activities, and (3) whether capacity growth has occurred in the developing country over the course of the collaboration (Additional file 1). Each interview lasted approximately 40 to 45 minutes. The interviews were recorded electronically using a tape recorder. The first author then transcribed them and this was cross-checked by the last author (VD). In order to protect anonymity, each transcript was marked with a unique case identification number and all names were removed. 

### Data analysis

The data collected were analyzed using thematic framework analysis. The transcripts were read and reread and initial codes were enlisted by research team. Each code was described briefly. From these codes, a list of categories were developed which were tabulated using Microsoft Excel 2010. This framework was applied again on the transcripts, charting the data onto excel, modifying categories where necessary. The final framework, consisting of 52 codes and 16 subcategories was analysed further to develop seven categories and three themes. To increase the reliability, the complete thematic framework was read by the last author independently and updated. Analyses were discussed repeatedly among the authors [27–29].

## Results

Three themes emerged from the analysis, namely (1) collaboration process: perception, phases and patterns; (2) communication and outputs hampered by Internet infrastructure and consortium size; and (3) outcomes of the collaboration: what was actually achieved (Table 2).

**Table 2:** Main themes, categories, subcategories and codes for understanding collaboration in a multi-national research capacity-building partnership

Codes	Subcategories	Categories	Theme
1. Capacity building 2. Network building 3. Resource generation 4. Passive partners 5. Unequal funding 6. Centralised role of Hub and Coordinating institutes	Perception about the project	Perception about the project: unequal participation depending on available funding	Collaboration process: perception, phases and pattern (Theme 1)
1. Adaptation of sister project 2. Funding availability	Idea behind the project		
1. Previous working ties 2. Similar aim and objective 3. Presence of expertise	Collaboration criteria	Collaboration process: importance of network	
1. Some old partners 2. New partners	Collaboration duration		
1. Organisational structure 2. Working flexibility 3. IT infrastructure 4. Knowledge and interest in subject area	Collaboration preference		
1. Collaboration among European partners 2. Collaboration among European and Asian partners 3. Collaboration among Asian partners 4. Collaboration within same country	Collaboration structure	Collaboration pattern: challenges between Asian partners	
1. Centralised working structure (with Hub and Coordinating institutes playing central role) 2. Little communication among small partners 3. Missing common goals	Limitation of collaboration pattern		
1. Technology-facilitated communication 2. Online meetings through Skype and Got meeting 3. Quarterly, annually and 18 monthly reporting 4. Monthly reporting 5. Emailing – most convenient method of communication	Communication process: methods used for communication	Email facilitating communication in a large consortium	Communication and outputs hampered by Internet infrastructure and consortium size (Theme 2)
1. Centralised communication (via: Hub and Coordinating institutes) 2. Overloading emails 3. Delayed responses by passive partners	Challenges		
1. Unavailability of full-time internet 2. Low band width, if available 3. Lack of Platform to conduct online courses, MOODLE	Inadequate IT infrastructure	Size of the consortium and diverse partners as a challenge to communication and activity	
1. Diverse partners with different languages 2. Need effective communication	Language problem while communicating		
1. Arranging online meeting is a challenge 2. Annual meeting – partner availability is a challenge	Time zone difference		
1. Tracking all ongoing activities in 13 partner institutes is a challenge 2. Employees, worked part time 3. Overburdened staff 4. Full focus on the project is the need 5. Less funding to small partners, fewer resources to use	Limited funds/other challenges		
1. Research capacity building is a gradual process 2. Capacity increased, but not significantly 3. Hub/Coordinating institute improved with mentoring activities and networking	Development in research capacity	Limited effects on research training capacity	Outcomes of the collaboration: what was actually achieved? (Theme 3)
1. Absence of indicators to map the gain 2. Process indicators, not sufficient to map the gain	Challenges mapping the gain		
1. Better online activities 2. Developed MOODLE 3. Developed IT infrastructure 4. Better human resource/project staff	Gain in other means	Infrastructure development as an additional outcome	

### Theme 1: Collaboration process: perception, phases and pattern

The collaboration process theme emerged from the following categories: (1) perception about the project – unequal participation depending on available funding; (2) collaboration process – importance of the network; and (3) collaboration pattern – challenges between Asian partners.

#### Category 1: Perception about the project – unequal participation depending on available funding

A clear understanding about the project aim and goal is important in an international collaborative project. It seemed that ARCADE RSDH partners shared an understanding of the project. Many interviewees saw ARCADE RSDH as a great way of extending collaboration and adding research capacity to their own institutes.

“*Our institute needs to work with other international institutes, to enhance our network*” *–* Interviewee, small partner organisation.

“*The peculiarity of this project is that the big partners have a bigger involvement, stronger roles and they tend to participate more. The involvement of smaller partners seems to be a little less involved, but there are some exceptions in our case*” – Interviewee, coordinating organisation.

Participants regarded the project as a way to improve both the collaboration capacity at the organisations as well as the educational capacity of the organisations.

#### Category 2: The collaboration process – importance of networks

The consortium was developed based on previous working experience. Initially, partners were selected based on previous working relationships with KIs and knowledge and interest in the subject area.

“*We knew that they were excellent from previous experience and we wanted to bring the collaboration to the new project to continue working with them*” – Interviewee, coordinating organisation.

Partner organisations were not just selected because they were known to the coordinator, they also had to have expertise and resources to implement the study. For example, one of the small partners did not have any previous relation with the coordinating institute, but joined the collaboration to extend its international collaboration.

“*No, this is the first time we are working with KI, we don’t have any previous collaboration with any of the institute. Our institute is very much aligned to the aim of the project and we need to enhance our network*” *–* Interviewee, small partner.

Similar interests with the project’s objectives acted as a motivation to join the project, though motivations differed between ‘smaller’ and ‘larger’ partners. Partners with less funding were motivated to participate by access to resources and opportunity to communicate with international experts, which would ultimately enhance their institute’s scientific/research carrying capacity. On the other hand, ‘larger’ partners wished to share their resources and skilful expertise with the developing institutes in order to help them gain technical capacity. This kind of mutual interest aided the collaboration:

“*KI approached us to give training to the less resourced research organisation and we have resources to help them, so we joined the project*” *–*Interviewee, big partner.

“*By this kind of collaboration, we could increase educational resource and communicate with international experts*” – Interviewee, small partner organisation.

#### Category 3: Collaboration pattern – challenges between Asian partners

The organisational culture and its structure influence any collaboration to a great extent. In this project, the preference for collaboration was with universities due to their flexible working style, though not all the partners were universities. According to one interviewee, the bureaucratic structure and other political issues often made collaboration difficult with some government run organizations because of their rigid working structure.

“*The role of politicians is very much higher so it is much more difficult to work with government institute especially in India. However, I am working with other institution in other countries, their political involvement is not there and it’s easy to work with them in comparison to India*” – Interviewee, coordinating institute.

While analysing the pattern of collaboration among ARCADE RSDH partners, collaboration was seen (1) between organisations in European countries; (2) between organisations in European and Asian countries; (3) between organisations in Asian countries; and (4) between organisations within the same country.

Active collaboration was seen between the European partners. One of the European partners is the coordinating organisation, which receives help from another European organisation in developing online courses.

The collaboration among Asian and European partners was seen as good, though there seemed to be less involvement of smaller Asian partners than the ‘larger’ Asian partners:

“*We have this kind of activity of mentorship. We have expert faculties from Chinese university and IDS [United Kingdom] *” – Interviewee, small partner organisation.

However, the links between the Asian partners were found to be weak. Only one or two institutes from Asian countries collaborated with each other as opposed to collaborating with European institutes. Some of the reasons mentioned for this included little interaction and previous experience of working together between partners. The differences in goals and administrative structures of the partners (medical colleges, management and research institutes, universities) also influenced the collaboration practice negatively.

“*We came to know our partner after the consortium was formed; most of the institutes are universities different from us so we don’t have much scope to learn from them*” – Interviewee, small partner.

Participants observed that ‘smaller’ partners collaborated with the hub institutes and other ‘larger’ partner organisations, but there was no or little interaction between them. The participants suspected this was caused by a lack of resources at ‘smaller’ partners, limited funding and differences in the educational system, language and culture of the institutes.

“*I think, as project needs more communication, many times we couldn’t get some difference in culture and language and we have different educational systems which poses challenges. Yes language is a big problem*” – Interviewee, small partner.

Missing common goals among the partner institutes may hinder the process of international collaboration. To some extent, this was the case in ARCADE RSDH – according to one of the small partner PIs, the programme intends to build the research capacity of the institutes by giving training to the PhD students and not the staff. Therefore, as these institutes did not have PhD students, they were not active participants.

### Theme 2: Communication and outputs hampered by Internet infrastructure and consortium size

This theme emerged from the following categories: (1) email facilitating communication in a large consortium, and (2) the size of the consortium and diverse partners as a challenge to communication and activity.

#### Category 1: Email facilitating communication in a large consortium

Technology plays an important role in building international collaboration. It helps to bridge the cultural diversity and differences in an international collaboration such as ARCADE RSDH. Expectations of the collaboration can be quite different, and therefore good communication channels between the partners are important, which is usually facilitated by technology. The most common and convenient method used in communicating daily activities was email. Technology such as Skype and GoToMeeting (www.gotomeeting.com) were used to arrange online meetings. While reports gave partners an idea of ongoing activities, the most interaction and sharing of work occurred at annual meetings:

“*This is a huge project so many people have joined and working in the same year, the best way to get more response is by email*” *–* Interviewee, small partner, Asia.

As the coordinating institute preferred to work via email, all partners also found that response from the coordinator was good.

“*We get loads of emails they are good at providing emails. They immediately reply. We come to know about all the activities*” – Interviewee, small partner, Asia.

Partners communicated in the consortium to different degrees – responses to the coordinator varied from partner to partner. Similarly, the hub institutes reported getting mixed responses from ‘smaller’ partners, as some replied promptly and others were more reluctant:

“*The main challenge is people don’t respond to emails so that’s the problem*” – Interviewee, hub institute, Asia.

#### Category 2: Size of the consortium and diverse partners as a challenge to communication and activity

The size of the consortium, and the diversity of partners, was one of the challenges in ARCADE RSDH. One of the main challenges is keeping track of all the ongoing activities, as ARCADE RSDH had 13 partners:

“*Since it is a big workload for the coordinator to keep track of all the activities of the partners, sometimes some of the activities go under the radar*” – Interviewee, coordinating institute.

Lack of in-depth knowledge of ongoing activities was also seen as a barrier to activity by small partners; as some partners were ignorant about the project activities they lost interest in the project and became passive:

“*We need effective communication we have so many partners. For example, I don’t know the recent development of this project and activities done*” – Interviewee, small partner organisation.

As some of the staff contributed to the project part-time, they were seen as overburdened. They could contribute only limited hours to the project, some due to limited budget. According to an interviewee, part-time involvement also affected the project outputs. One's entire attention is needed on the project to make the project successful. Inability to motivate the partners was also stated as a shortcoming. 

“*If we want ARCADE to happen really the interest should be solely focused on ARCADE activities. Motivation shall be as such that one really pays attention to this. Now the communication between the organisations is also must, the annual meeting is also a problem as not all are able to participate because of the time difference or the difficulty to reach the place. Having a conference on Skype is also difficult*” – Interviewee, big partner organisation.

The basic structure of the consortium, in which the hub institutes communicated with smaller partners and coordinating institutes, was seen as one of the challenges in communication. This specific structure sometimes resulted in turbulent flows of information as the exchange of information mostly occurred among the coordinating institute and larger partners and less between small partners. Smaller partners were mostly dependent on the hub institute for communication:

“*Another problem is the structure of ARCADE is based on hub working/communicating with small institutes in some way that has not been working and this is creating a problem*” – Interviewee, coordinating institute.

According to the participants, new ways should have been explored to engage all the partners in the project. To increase their involvement, they should be told about the benefits of engaging themselves in grant writing and grant management workshops, which will later help them build their research capacity. The diversity of partners meant that there were several challenges to address in communication, including lack of infrastructure for communication and time differences.

#### Lack of infrastructure as barrier to collaboration

Within the large consortium, lacking infrastructure, including internet facilities in ‘small’ partner organisations, affected the planned ongoing activities of the project. Even if Internet was available, the bandwidth may not have been sufficient, which was one of the most important prerequisites for conducting online courses and other online activities for a project like ARCADE RSDH. All this resulted in a weak collaboration, especially among the small partners and between small and big partners.

“*If you want to give good video or audio, bandwidth shall be good, but the institute is not able to give its entire bandwidth to this education*” – Interviewee, coordinating institute.

As can be expected, no technical issues were reported in European partners.

#### Time difference

As many of the partners were from different countries, time differences also hampered the collaboration and resulted in increased dependency on the use of email. Arranging online meetings with all the partners was difficult as any time decided was odd for one or another partner.

“*Setting up of some meeting, which would engage one person from Canada and other partners based in China and Vietnam, bringing up everyone in the same meeting is challenging*” – Interviewee, coordinating institute.

The small partners, who were less active, did not find timing as a major issue in collaboration.

“*Until now, we have not arranged any such meetings so I don’t see any challenge, it’s just a management issue*” – Interviewee, small institute.

### Theme 3: Outcomes of the collaboration: what was actually achieved?

This theme emerged from the following categories: (1) limited effects on research training capacity and (2) infrastructure development as an additional outcome.

#### Category 1: Limited effects on research training capacity

Participants felt that building research capacity for any institute is a gradual process and takes time, though enhancement in the research capacity was certainly observed.

“*Increase in research carrying capacity, specifically in SDH* [social determinants of health] *is not that significant, if judged by the no of proposals submitted for funding. But it will soon become better. I cannot score it high*” – Interviewee, small partner organisation.

Hub institutes felt that their capacity had increased, with mentoring activities, development and networks being extended:

“*There are courses which are not developed in our institute but are developed by other partner organisations, so our students get the benefit of those courses. Other than that it’s an internal learning too, how to work in collaboration with other organisations*” – Interviewee, big partner organisation, European country.

According to participants, mapping the impact of research capacity building activities was essential to an international collaborative project. For this, certain indicators were to be developed. ARCADE RSDH had some process indicators to assess the ongoing activities in the project, but those were not sufficient to map the gain in the research carrying capacity of the partner institute.

“*This is something we haven’t really captured with indicators as to how our project is progressing and this is very much qualitative and depends on the level of satisfaction, etc*.” – Interviewee, coordinating organisation.

#### Category 2: Infrastructure development as an additional outcome

Some participants reported that they had developed in terms of infrastructure, which they were lacking earlier, and had become increasingly aware about grant management and implementation of online activities such as meetings and courses. An increase in human resource capacity was also observed in partner organisations. Specific platforms for online learning, such as Moodle (www.moodle.org) were adopted and developed by the hub institutes to conduct online courses. Many blended courses had been conducted across the partner institutes and a good enrolment of students for these courses was seen.

“*Staff has increased so small partner organisation’s capacity has also grown. I think last time, when online activities were conducted, they were not that good as of now. Things are better technically* [Internet connectivity]” – Interviewee, hub institute.

## Discussion

This study explored the process of collaboration in an international collaborative project, ARCADE RSDH, which focused on building research capacity of young postgraduate, doctoral and postdoctoral researchers. The project activities were carried out in a collaborative environment, with different collaborative partners and a unified goal [30]. The project partners were assigned different roles that ranged from providing the resources to being the end-users of materials developed within the project. The study highlighted how a collaboration begins and develops, and the challenges and solutions for communication and achieving the set goals.

Collaborative projects are often seen challenging, as the project managers come from different organisations and backgrounds to work together. The diversity in cultural and national backgrounds, skill sets and expertise, and managerial roles, results in different expectations, which may be difficult to meet [30].

ARCADE RSDH had 12 partners with diverse cultural and organisational backgrounds, which were brought together mainly due to previous working relationships. It is often seen that human, linguistic or social ties form as a result of historical interactions and support present-day collaborations [14, 18]. The partners involved in ARCADE were known to each other through previous work relations, though not all of them were familiar with each other. These working relationships were not necessarily long term.

Other studies have suggested that spatial proximity encourages collaboration [14]. ARCADE RSDH did not seem to be influenced by spatial proximity as much as previous working ties. However, the geographic spread also created challenges in collaboration, including time zones, organisational cultures and distance to meetings despite an active use of online resources. In order to counter the effects of distance, emails were used as the main mode of communication. However, email is usually overloaded and often missed by project staff [14, 18], and thus may not be the most effective mode of communication [14]. Project-related communication was enhanced by quarterly and annual meetings, but not all partners attended these. In order to overcome these communication challenges a robust communication strategy should be planned, which should encourage face-to-face interaction among all the project partners, taking into account the time and cultural differences in the beginning phase of the project [22, 29].

One of the barriers found in this project, both with regards to communication and achieving outcomes, was understanding each other’s infrastructures and administrative organisations. In this collaboration, some partners who initially committed to the project later became passive. This could have been due to incentives not aligning with the demand for achievements, recognition and rewards [22, 31]. Lack of a clear understanding about other partners’ organizational structure and working style is also one of the reason behind them becoming passive. Successful collaborations require an understanding of each other’s working environment from the project planning stage of the partnership in order to get a clear insight about each other’s working methods. This alignment can be achieved by openly discussing the differences and processing them at the beginning of the project, enhancing a focus driven collaboration [32].

Paradoxically to the aim of collaborations to enhance research capacity, existing research capacity in an institute also attracted collaboration with international partners in our study. Research indicates that scientific research requires a ‘baseline’ level of scientific infrastructure to make collaboration effective in capacity building [14]. Therefore, institutions that do not have capacity at that critical level may not attract further capacity building, an issue that deserves attention and further research.

One reason for “smaller” partners becoming passive could be the funding structure and management structure that operated through hubs [33]. In another collaboration, African partners could not contact each other directly and were obliged to direct their communication through United Kingdom-based researchers [6, 34, 35]. This kind of structure can decrease communication among the partners, and thus result in passivity. Attention should also be paid to funding structure in projects. ‘Small’ partners with limited resources cannot commit resources, including staff, to projects fully. Paying attention to ‘smaller’ partners’ needs is important in planning activities. This helps enhance their research capacity, and in ensuring an equitable approach to capacity building [36].

Long and sustained collaboration is key to a project’s positive outcome. Sustained, systematic effort is particularly needed in collaborations aiming to build research capacity [8]. Short-term collaborations often lead to loss of existing achievements, especially in LMICs, with capacities left unused and researchers migrating away in search of other job opportunities [13]. Research projects receive funding for a short period, which allows the creation of the project, but sustaining it longer when timelines are strict and further financing limited is challenging [37]. ARCADE RSDH was a 4-year span project, which received its funding from the European Union Seventh Framework Programme. These activities came to an end after 4 years, though relationships were developed with project partners. Our participants felt that collaboration in ARCADE RSDH had indeed helped to develop research carrying capacity of partner organisations in various ways, though the activities initiated should have been carried on further as research capacity building takes many years and systematic effort and is not built in a short time frame.

### Limitations

This study explored international collaboration using a cross-sectional, qualitative study. As the study was conducted at a particular point of time during the middle phase of the project, it cannot represent the working of the collaboration during the entire project. Following the implementation from the beginning to the end may have yielded more substantial results. This study did not assesses the process indicators and outcomes of activities planned during the project. Evaluation of such indicators in future will give more insight of research collaboration in such a large consortium.

## Conclusion

This study explored work in a large, North–South collaboration project focusing on building research capacity in partner institutes. Collaborative partners emphasised the need to set up clear targets, communication strategies and align research interests at the start of collaboration. Managing these challenges requires pre-planned strategies to develop proper communication channels among the partners, maintaining transparency, and sharing the rewards and benefits at all stages of collaboration. North–South collaborations should ensure that funding is equitable to ensure active participation. Collaborations such as ARCADE RSDH have potential to build research capacity in all partners, but such activity needs sustained and systematic effort.

## Abbreviations

ARCADE RSDH, Asian Regional Capacity Development Research on Social Determinants of Health; LMIC, low- and middle-income countries

## Additional file

Additional file 1: Topic Guide – Understanding collaboration in a multi-national research capacity-building partnership. (DOCX 18 kb)
