# Supplementary material for: Understanding collaboration in a multi-national research capacity-building partnership: a qualitative study
Source: Health Res Policy Syst. 2016 Aug 18;14:64. doi: 10.1186/s12961-016-0132-1 (PMC4991081; doi:10.1186/s12961-016-0132-1)
Supplement: Additional file 1: — Topic Guide – Understanding collaboration in a multi-national research capacity-building partnership. (DOCX 18 kb) [file 12961_2016_132_MOESM1_ESM.docx]

**Supplementary File.**

**Topic Guide - Understanding collaboration in a multi-national research capacity building partnership**

| - What is your perception about the project ARCADE? - When did your organization joined this project? - Is this the first time you are working with the coordinating institute, KI? - Do you know your other collaborators/ have previous work experience with them? - Why did you choose to collaborate in this project? - How this collaboration has enhanced your organization’s research capacity? - How do you exchange your information with collaborating organization? - What are the challenges you face in this collaboration? |
| --- |
